# Analysis of surgeon biometrics during open and robotic radical cystectomy with electromyography and motion capture analysis

**DOI:** 10.1590/S1677-5538.IBJU.2019.0163

**Published:** 2020-01-13

**Authors:** Adam Baumgarten, Joon Kyung Kim, Jeff Robison, John Mayer, Dustin Hardwick, Trushar Patel

**Affiliations:** 1 Department of Urology, University of South Florida, FL, United States; 2 Department of Physical Therapy, University of South Florida, FL, United States

## Abstract

**Purpose:**

To determine feasibility of measuring surgeon physical stress during both open radical cystectomy (ORC) and robotic radical cystectomy (RRC).

**Materials and Methods:**

One patient underwent ORC, while the other underwent RRC by a single surgeon. The diversion was excluded from this study. Noraxon^®^ myoMOTION^™^ kinematics sensors were used to quantify the amount of joint and segmental motion of the spine, shoulders, and head. myoMUSCLE^™^ EMG sensors were used to measure activation levels, patterns, and fatigue characteristics of key muscle groups. The Prone Static Plank Test (PSPT) and Modified Biering-Sorensen Test (MBST) were used to assess surgeon strength and endurance of core musculature.

**Results:**

The surgeries were represented in five stages. During ORC, the percentage of time spent in cervical flexion was 98%, 91.8%, 87.5%, 100%, and 97.1%, respectively. During RRC, 100% of the time was spent in cervical flexion. Activation of key muscle groups was examined across all stages and expressed as a percentage of peak activation. MBST times were both 25 second pre-and post-surgery ORC and 25.1 seconds pre-surgery and 32.4 seconds post-surgery for RRC. PSPT times were 68 second pre-surgery and 48 seconds post-surgery for ORC, and 59 second pre-surgery and 51 seconds post-surgery for RRC.

**Conclusion:**

We were able to identify meaningful data using kinematic and EMG analysis during ORC and RRC. We were able to identify target muscle groups that will be used to conduct a larger study with multiple surgeons to help determine if there is an ergonomic advantage to RRC over traditional ORC.

## ARTICLE INFO

**Available at: http://www.intbrazjurol.com.br/video-section/20190163_Baumgarten_et_al**

**Int Braz J Urol. 2020; 46 (Video #1): 138-138**

